# Current trends in endovascular management of intracranial aneurysms (including posterior fossa aneurysms and multiple aneurysms)

**DOI:** 10.4103/0971-3026.41841

**Published:** 2008-08

**Authors:** Santhosh Joseph, Ravindra Kamble

**Affiliations:** Department of Radiology, Sri Ramachandra Medical College, Chennai - 600 116, India

Aneurysmal subarachnoid hemorrhage (SAH) is a devastating illness and a major cause of morbidity and mortality.[1] The frequency of aneurysm ranges from 0.4-10%; there is a prevalence of 2.3% in adults without a risk factor for SAH and an annual risk of rupture of 1.9%.[2] Once the aneurysm has bled, there is 15% chance of rebleed in the first few hours, and this is associated with high mortality.[3] Therefore, intracranial aneurysms (IAs) has to be treated surgically or endovascularly. With the publication of the International Subarachnoid Aneurysm Trial (ISAT) results, more and more aneurysms are being treated by endovascular means worldwide.[4] However, each aneurysm should be assessed by an interdisciplinary approach, with the aim being to choose the best option for each patient. Advanced techniques in endovascular therapy have made this modality relatively safe and as effective as surgery in experienced hands.

## Endovascular Treatment of Intracranial Aneurysms

Endovascular therapy can be basically deconstructive or reconstructive, depending on whether the parent artery has to be preserved or not.[2] Historically, ruptured aneurysms were treated by electrothermic thrombosis or by piloinjection.[2,5] A new era in the treatment of IA began when Italian neurosurgeon Guido Gugliemi introduced electrolytically detachable platinum coils in 1991.[6] Today the focus is on measures to promote endothelialization across the aneurysm neck after coiling in order to prevent regrowth and rebleeding. Proper preoperative and postprocedure care is however essential for reducing mortality and morbidity and achieving good patient outcomes.

## Preprocedure Workup, Technique, and PostProcedure Care

All SAH management is done in the critical care unit with the aim of providing complete rest to the patient and avoiding all external stimuli to prevent an adrenergic response. The patients should be put on antispasmodics, antiepileptics, and supportive treatment with monitoring of vitals. All important blood investigations should be done. Transcranial Doppler (TCD) can also be done to assess vasospasm.

## Technique and the Procedural Care

Endovascular treatment of aneurysm is done under general anesthesia. Baseline Activated Clotting Time (ACT) is measured and thereafter heparin infusion/bolus is given to maintain ACT at roughly 3 times the baseline throughout the procedure. Then a guiding catheter (Envoy, Cordis) is placed in the distal internal carotid artery (ICA) to obtain a stable position. With the help of a suitable microcatheter and microguidewire, the aneurysm is catheterized and subsequently coiled. At the end of coiling, an image is acquired to confirm adequate coiling. Supplementary doses of antiepileptics, steroids, and antiplatelet drugs are given after the procedure. A postprocedure CT is done if required.

## Postprocedure Care

Postprocedure care is done in the critical care unit, with close monitoring of all vitals. The patient is kept on heparin infusion for 12 h to keep the ACT 2-2.5 times above baseline value and is followed by low molecular weight heparin, 2500 U sc, for 24-48 h. Clopidogrel and low-dose aspirin are also given for at least another 3 months. The patient is kept on positive fluid balance. Following the procedure, to prevent vasospasm the patient is kept on nimodipine with continuous monitoring by TCD.

## Follow-up

Patients are advised to come for follow-up visits at the end of 1, 3, 6, and 12 months, and yearly thereafter; angiogram is advised after 6 months to assess the status of the coiled aneurysm. Patients are kept on nimodipine for 1 month and clopidogrel and aspirin for at least 3 months to prevent delayed ischemic neurological deficits.

## Embolic Materials Used in Treating Intracranial Aneurysms

Materials used in the treatment of IAs are coils, liquid embolic agents, and stents.

### Coils

Various coils are available in the market with different shapes and sizes and of different materials.

**Figure 1 (A, B):**
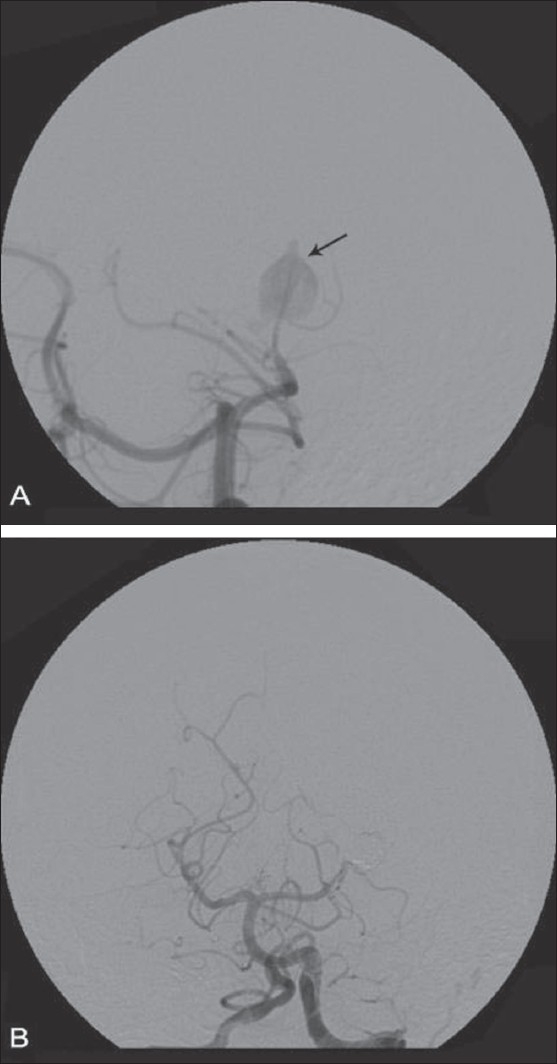
Left PCA (P3 segment) aneurysm coil embolization. Angiogram images showing preprocedure PCA aneurysm (arrow in A)and post-coiling status (B)

1. *Electrolytically detachable platinum microcoils*: Guglielmi detachable coils (GDCs; Target Therapeutics) are platinum coils soldered to the end of an insulated stainless steel guidewire; they can be detached by passing a direct current through the wire; there is also the advantage that the coils can be retrieved if necessary. These coils are available in various sizes and shapes, e.g., GDC-10 or GDC-18 in standard, soft, and ultrasoft types. Coils are available in 2D, 3D, and complex 3D shapes. A variety of coils are available, with varying degrees of softness and of different shapes, as well as coils with thrombogenic filaments and drug coatings. The detachment technique also varies and can be electrolytic, hydrophilic, thermal, or mechanical [Figure 1].
2. *Hydrocoils (MicroVention)*: This group of coils has a coating of synthetic polyalcohol over the platinum coil which can swell upto 8-9 times within 6-8 min on contact with blood, causing good volumetric packing. These coils are also designed to permit reposition, retrieval, and detachment.
3. *Bioactive coils*: The main purpose of developing bioactive coils is to produce an enhanced cellular response, which would stimulate neointima formation and provide a good endothelial lining across the aneurysm neck, thus preventing regrowth and rebleeding. The various types of bioactive coils available for aneurysm management are, for example, a) matrix coils, b) coils coated with extracellular matrix, c) coils coated with cytokines and or growth factors, d) Cerecyte (Micrus) and Nexus (EV3), and e) gene delivery microcoils. Of these, matrix coils are being increasingly used in clinical practice since they provide better inflammation and coil compaction compared to hydrocoil and platinum coils.[7]

### Liquid embolic agents

**Figure 2 (A-C):**
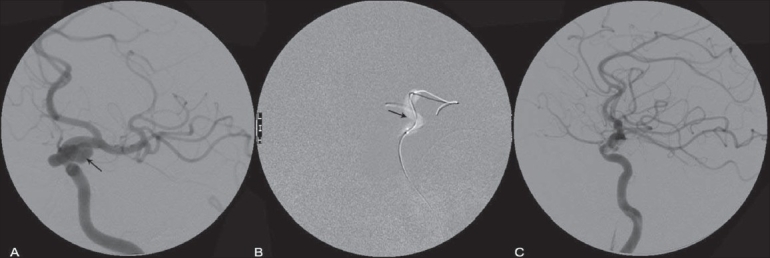
Onyx embolization of ICA aneurysm. Angiograms showing the ICA aneurysm (arrow in A), balloon inflation across the aneurysm (seal test) (arrow in B) and post-onyx embolization (C)

*Onyx (HD 500)* : It is a biocompatible polymer (ethylene-vinyl alcohol copolymer, EVOH) dissolved in organic solvent dimethyl sulfoxide (DMSO), with micronized tantalum powder added to obtain adequate radiopacity. Seal test with contrast is required to confirm leakage into the parent artery before injecting onyx. This mixture requires dedicated DMSO-compatible microcatheters (Ultraflow or Rebar) to prevent material incompatibility between solvent and hub plastics. In 2004, CAMEO (the Cerebral Aneurysm Multicenter European Onyx) trial was carried out to study the feasibility and results of onyx injection in intracranial wide-neck aneurysms. The study showed good results of onyx injection in wide-neck aneurysms and in those otherwise unsuitable for coiling.[8] However, the use of onyx is currently limited to large and giant aneurysms below the posterior communicating artery (PCOM) location [Figure 2].

### Intracranial stents

Various intracranial stents are used for treating aneurysms, either alone, or as stent-assisted coiling/embolization with onyx or a mixture of both coils and onyx, especially in aneurysms with a wide neck (a dome/neck ratio of less than 2.0 and a neck diameter of more than 4-5 mm) to prevent recanalization. Various types of intracranial stents are available, e.g., Neuroform stent (Boston Scientific), Leo stent (Balt), self-expanding Nitinol stent (Cordis Enterprise), and electrolytically detachable SOLO stents. Leo and Cordis Enterprise stents are of closed-cell design and Neuroform stents are of open-cell design. The closed-cell design allows all coils to be placed within the aneurysm and outside the flow of the parent artery with stent-assisted coiling.

Neuroform stents have the disadvantage of being non-retrievable and strut prolapse can occur in curved vasculature due to its open-cell design.[9] Both Leo and Enterprise stents can be retrieved or repositioned even after 90% of deployment; this allows safer and more precise deployment.[10]

**Figure 3 (A-C):**
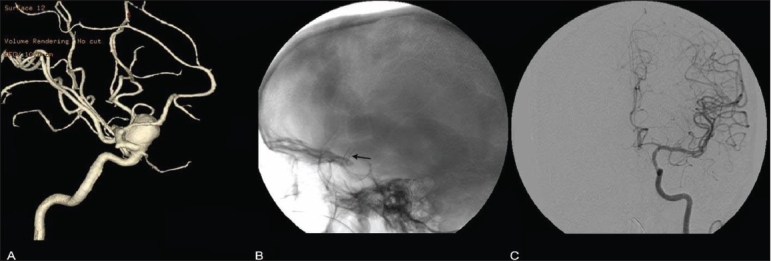
Stent-graft (cardiac covered stent) for a large ICA aneurysm. 3D rotation angiogram (A) shows an ICA aneurysm (aneurysm).A plain skull lateral radiograph shows the stent (arrow) across the aneurysm. Angiogram (C) shows post- stent non-filling of the aneurysm

Specially designed covered stents for intracranial vasculature have been used for the management of pseudoaneurysms in the intracranial ICA[11][Figure 3].

## Novel Techniques Used in the Treatment of Intracranial Aneurysms

### Balloon remodeling technique

This technique has basically permitted endovascular treatment of IAs with an unfavorable dome-to-neck ratio (wide neck).[12] Hyperglide or hyperform balloons can be used for this technique. The balloons can be placed across the neck of the aneurysm, to be inflated and deflated intermittently during coiling of the aneurysms to prevent coil prolapse.

**Figure 4 (A-C):**
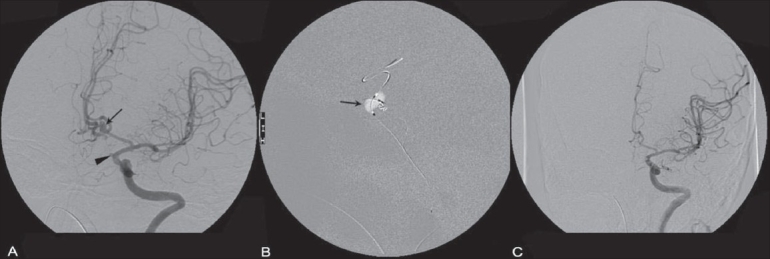
Balloon assisted coiling of the ICA and ACOM aneurysm. Angiogram images show the ACOM (arrow) and supraclinoid ICA (arrowhead) aneurysms (A), balloon inflation across the ACOM (arrow in B) and the post-procedure status (C)

- *Single balloon remodeling technique*: The advantages of balloon-assisted coiling (BAC) are: (1) ‘control’ of blood flow in the vessel; (2) improved stability of the microcatheter in the aneurysm; (3) denser packing with coils; and (4) improved delineation of the neck of the aneurysm. Although complications are associated with its use, the risk/benefit ratio is favorable in BAC.[13] Further, in the event of intraprocedural rupture, balloon is a very effective tool to manage an acute crisis. In wide-neck aneurysms, bifurcation aneurysms, basilar top aneurysms, and giant aneurysms, this technique of BAC appears to be very useful [Figure 4].
- *Double balloon remodeling technique*: This technique is useful in bifurcation aneurysms (MCA) and in wide-neck aneurysms distal to the circle of Willis. Two compliant balloons (hyperform) can be placed across the neck in two branches of the artery (superior and inferior division of the MCA in MCA bifurcation aneurysm or in a crossed X configuration, from A1 of one side to A2 of the other side, in anterior communicating artery (ACOM) aneurysms) to cover the entire neck and prevent coil prolapse. The advantage of this technique is that it permits treatment of wide-neck aneurysms, and side branch protection and sealing of the neck can be better than with the single balloon technique. However, the disadvantages include thromboembolic complications due to the use of multiple catheters and technical difficulties.[14]

### Stent remodeling technique

**Figure 5 (A-C):**
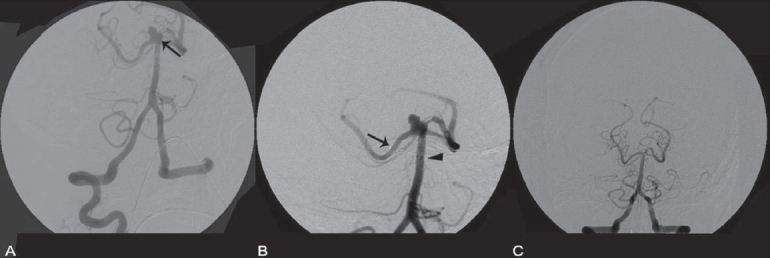
Stent-assisted coiling for a basilar apex aneurysm. Angiogram images show a basilar apex aneurysm (arrow in A), a stent across the aneurysm with one end (arrow) in the right PCA and the other (arrowhead) in the basilar artery (B) and the post-procedure status

- *Single stent*: The stent is placed across aneurysm neck and a microcatheter is passed through the struts into the aneurysm for subsequent coiling. Reconstructive treatments using stents improve occlusion rate and protect parent vessels. The presence of the stent will also change the hemodynamics in the aneurysm lumen, leading to thrombosis. This technique can be used in all types of complex, wide-neck, saccular aneurysms at any location (but especially in the basilar tip and the carotids), in fusiform aneurysms, dissecting aneurysms, pseudoaneurysms, and giant aneurysms[15][Figure 5].
- *Double stent technique*: In case of large fusiform aneurysms, two overlapping stents help in vascular remodeling, with stent endothelialization and patent parent artery flow and thrombosis of aneurysm.[16] Wide-neck bifurcation aneurysms (e.g., MCA bifurcation or basilar tip) can also be treated with the use of two stents in two branches, in a ‘Y’ configuration, to provide protection from coil prolapse in the parent artery.[17]

## Management Strategies with Different Types, Locations, and Morphology of Intracranial Aneurysms

### Management of ruptured saccular aneurysm with a narrow neck

Narrow-neck saccular aneurysms can be treated by using any of the coils described above. Optimum results with coiling (used alone) can be obtained when the dome-to-neck ratio is at least 2.[18] For aneurysms treated with GDC, a complete obliteration rate of 61% and subtotal obliteration rate of 22% at follow-up after more than 6 months, has been described.[19] However, follow-up angiography for a longer period is needed after endovascular treatment. After GDC, 25% of neck remnants exhibit progressive thrombosis and 49% show recanalization, on follow-up angiography, especially in wide-neck and large/giant aneurysms. However, there has been a decrease in the rate of recanalization in recent years, and with the advent of new remodeling techniques, the treatment of wide-neck aneurysms is no longer as problematic as it used to be.[20]

### Management of ruptured saccular aneurysms with wide neck

Treatment of wide-neck aneurysms differs slightly from that of narrow-neck aneurysms and is more challenging. The major problem is of coil prolapse and migration. To avoid this, remodeling techniques are used, such as stent-assisted and balloon-assisted coiling, which provide a stable coil mass and prevent prolapse. Another method of treating wide-neck aneurysms is by embolization with the use of a liquid embolic agent like onyx (HD 500), either alone or in combination with coils.[21]

### Management of unruptured saccular aneurysms

With regard to the treatment of an unruptured aneurysm, although the options remain the same, there is controversy over whether these aneurysms should be treated at all. Various factors has to be taken into considerations while addressing unruptured aneurysms, for example, the age of the patient, the clinical presentation, the size and location of the aneurysm, and its chances of rupture. The rate of rupture of unruptured aneurysms of less than 10 mm size is 0.05% per year and for those more than 10 mm in size the rate is < 1 % per year. For those patients presenting with SAH from another aneurysm, the risk of rupture is 0.5% per year.[22] In another study by the same group, rupture rates were found to be higher in posterior circulation aneurysms than in anterior circulation aneurysms of the same size, with rupture rates increasing with size of aneurysm.[23] Thus, a conservative approach to treating unruptured aneurysms may not be useful; they should be treated to prevent rupture, taking into account factors like age, clinical presentation, and the number of aneurysms. Compared to conventional neurosurgical management, endovascular treatment appears to be the better option for treating unruptured aneurysms due to its favorable patient outcome in the form of less morbidity and mortality and reduced hospital stay.[24,25] Thus, it has been recommended that surgical treatment of an unruptured intracranial aneurysm in patients over age 50, and any treatment of patients over age 70, should be approached with particular caution.[26]

### Management of fusiform and dissecting aneurysms and pseudoaneurysms

These types of aneurysms can be better treated by parent artery occlusion or by use of stents. Sometimes, only stent deployment across the fusiform aneurysm will alter the flow hemodynamics and cause obliteration of the aneurysm.[16] Stent placement and/or stent-assisted coiling can be better alternatives than parent artery occlusion in treating dissecting aneurysms.[27] Stent-assisted coiling is also now a novel therapy in the treatment of circumferential (fusiform) aneurysms, where any other endovascular treatment method is likely to fail.[28] Cardiac covered stents can be used for treating carotid pseudoaneurysms.[29]

### Management of blood blister-like aneurysms (BBA)

These are small aneurysms, most commonly arising from the ICA. They can cause fatal SAH. On pathology, BBA are seen as lacerations on the vessel wall due to internal elastic lamina degeneration.[30] These aneurysms can be treated by coiling.[31] Balloon-assisted coiling may be useful for these small aneurysms.

### Management of giant and large intracranial aneurysms

Giant aneurysms, defined as those larger than 25 mm, constitute 5-8% of aneurysms. Sixty percent occur in the ICA, the cavernous portion being the most common site. They present most commonly with mass effect (75%), thromboembolism, or intracerebral hemorrhage. Surgical proximal trapping is considered the best option. Endovascularly, giant aneurysms can be treated by using just coils/stent, stent or balloon-assisted coiling, covered stents with parent artery preservation, or parent artery occlusion, or onyx. Weber *et al*, have used onyx in large and giant aneurysms with wide necks and obtained complete obliteration in 91% of cases, without any permanent severe morbidity and no mortality.[32] Long-term follow-up studies of parent vessel occlusion as a treatment for giant and large aneurysms have found that the method could provide persistent exclusion of aneurysms with good clinical tolerance.[33]

### Management of mycotic aneurysms and peripheral aneurysms

Mycotic aneurysms account for 2-3% of all intracranial aneurysms. Septic emboli from the vegetations of bacterial endocarditis are the main cause in its formation (5%). These aneurysms are often multiple and irregular, situated more distally on peripheral branches, and have high chances of rupture. Treatment options include antibiotics, surgery, and endovascular coiling or parent vessel occlusion.[2,34] Peripheral aneurysms can be treated by coiling and/or parent vessel occlusion using glue.[35]

### Management of posterior circulation aneurysms

Treatment of posterior circulation aneurysms poses great technical challenges to the neurosurgeon. Endovascular, treatment of these aneurysms is much easier and is associated with lower mortality and morbidity.[2] The advent of remodeling techniques has revolutionized the treatment of posterior circulation aneurysms, especially of wide-neck basilar apex aneurysms. The use of the balloon/stent remodeling technique can lead to effective occlusion of aneurysms in the basilar apex, as described above [Figure 3]. In the series of Pandey *et al*, more than 95% dome occlusion of aneurysms was seen in 87.8% cases. At follow-up, 24.5% cases showed recanalization of at least 5%, requiring retreatment. Thus endovascular coil embolization of posterior circulation aneurysms is an effective treatment in the short term, but over the long term, retreatment may be necessary due to recurrence.[36] In another series, introduction of 3D GDCs was associated with an improved initial occlusion rate, but it did not affect the recanalization rate and there was a morbidity of 4.3%.[37]

### Management of aneurysm in elderly patients

Management of aneurysms in the elderly is a difficult task due to associated procedural risks and comorbid conditions. In the study done by Luo *et al*, in ruptured cerebral aneurysms in elderly patients, endovascular treatment with coil embolization and stent-assisted coiling was found to be safe.[38] It was found that elderly patients presenting with SAH with a low Hunt and Hess grade showed a better outcome than those with a high Hunt and Hess grade.[39] In unruptured aneurysms in the elderly, life expectancy and risk of rupture should be estimated for each patient individually before taking the decision to treat. Taylor *et al*, reported that aggressive treatment may not be beneficial as 2% of unruptured aneurysms in elderly patients rupture within 2.5 years of diagnosis.[40]

### Management of multiple aneurysms

The frequency of multiple aneurysm ranges from 5-33% and is more common in females than males.[41] The treatment of multiple aneurysms should always take into consideration the age of the patient, the neurological status, and the anatomic location of the symptomatic aneurysm. In case of multiple aneurysms, it is always important to locate the aneurysm, which has ruptured. The ruptured aneurysm must be treated first, with the other unruptured ones being treated later or in the same sitting. There are few criteria to identify the symptomatic aneurysm. Location of the blood on a CT scan helps to identify a symptomatic aneurysm. The larger and more irregular aneurysm is usually the one that has ruptured. If there are two aneurysms in one artery, it is usually the most proximal and larger aneurysm that has ruptured.[2] Nehls *et al*, have shown that in multiple aneurysms, there is a 97.5% possibility to correctly localize and identify the ruptured aneurysm, using CT scan, angiography, and clinical findings.[42] In comparing management outcomes after one year of multiple aneurysms *vs * single aneurysms, with the same Hunt and Hess grade, it was found that patients with multiple aneurysms had poorer outcomes (29%) compared to those with single aneurysms (19%).[43] The major advantage of endovascular treatment is that multiple aneurysms can be treated in a single session, whereas surgical clipping of multiple aneurysms in a single approach is difficult. Solander *et al*, in evaluating their results of treating multiple aneurysms as a single-stage procedure, concluded that endovascular GDC treatment of multiple cerebral aneurysms, regardless of their location, can be performed safely in one session, with excellent clinical outcome in 89%. By treating multiple aneurysms the chances of rebleeding as a result of having mistakenly treated another aneurysm, are also eliminated.[44]

### Complications of endovascular treatment and their management

Procedural complications induced by treatment itself are mainly thromboembolic events and aneurysm rupture. Procedural morbidity ranges between 3.7-10% and mortality between 0-2.1%.[2] MRI after endovascular treatment may show new hyperintense lesions, suggesting the occurrence of thromboembolic events. This group has suggested that antiplatelet treatment be given before and during the intervention procedure, in addition to heparin, to prevent thromboembolic complications.[45]

Intraprocedural rupture during embolization can occur due to microguidewire, microcatheter, or coil; it is a rare but unavoidable and life-threatening event and heparin should be immediately reversed by protamine sulphate. The majority of patients with an intraprocedural ruptured aneurysm will survive without severe sequelae, if managed appropriately.[46] Another feared complication during endovascular treatment is coil prolapse. Pulsating coil prolapse is associated with a high chance of coil migration, which can lead to thromboembolic complications. In this situation one of the following may be done; the coil can be retrieved with the help of a microsnare or pushed back with the balloon alone, or ignored, while adding antiplatelet therapy to the patient's regimen.[47] Parent artery or branch artery occlusion due to thromboembolism during the procedure can be tackled with the use of rTPA or abxicimab, with 81% improvement in TIMI grade.[48] Vasospasm is an important factor in determining the postprocedure outcome of the patient and therefore it has to be treated aggressively, with continuous monitoring. Vasospasm can be treated by triple-H therapy, intraarterial nimodipine/nicardipine, and angioplasty.[49,50]

Thus, with the advancement in endovascular techniques, IAs at any location, of any morphology, of any grade, and occurring at any age can be effectively treated with acceptable mortality and morbidity
